# Computational Evidence of NAGNAG Alternative Splicing in Human Large Intergenic Noncoding RNA

**DOI:** 10.1155/2014/736798

**Published:** 2014-06-05

**Authors:** Xiaoyong Sun, Simon M. Lin, Xiaoyan Yan

**Affiliations:** ^1^Agricultural Big-Data Research Center, College of Information Science and Engineering, Shandong Agricultural University, Taian, Shandong 271018, China; ^2^Biomedical Informatics Research Center, Marshfield Clinic Research Foundation, Marshfield, WI 54449, USA; ^3^Affiliated Hospital of Shandong University of Traditional Chinese Medicine, No. 42 Wenhua West Road, Jinan, Shandong 250011, China

## Abstract

NAGNAG alternative splicing plays an essential role in biological processes and represents a highly adaptable system for posttranslational regulation of gene function. NAGNAG alternative splicing impacts a myriad of biological processes. Previous studies of NAGNAG largely focused on messenger RNA. To the best of our knowledge, this is the first study testing the hypothesis that NAGNAG alternative splicing is also operative in large intergenic noncoding RNA (lincRNA). The RNA-seq data sets from recent deep sequencing studies were queried to test our hypothesis. NAGNAG alternative splicing of human lincRNA was identified while querying two independent RNA-seq data sets. Within these datasets, 31 NAGNAG alternative splicing sites were identified in lincRNA. Notably, most exons of lincRNA containing NAGNAG acceptors were longer than those from protein-coding genes. Furthermore, presence of CAG coding appeared to participate in the splice site selection. Finally, expression of the isoforms of NAGNAG lincRNA exhibited tissue specificity. Together, this study improves our understanding of the NAGNAG alternative splicing in lincRNA.

## 1. Introduction

**Figure 1:**
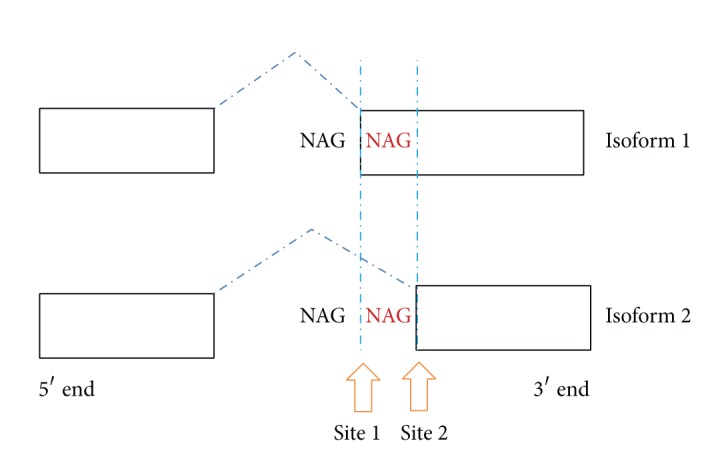
NAGNAG alternative splicing can result in two isoforms. The NAGNAG acceptors at the 3′-end can be either at site 1 or site 2, are three nucleotides apart, and exhibit the “NAGNAG” motif signature.

The NAGNAG alternative splicing mechanism is a process which facilitates alternative protein expression from a single gene. Analysis of deep RNA-sequencing data by Bradley et al. (2012) confirmed that NAGNAG is highly regulated [1]. NAGNAG alternative splicing specifically targets inclusion or exclusion of three nucleotides at 3′ splice sites (Figure 1), thus effecting a change in one or two amino acids encoded in the final protein [2–9]. Such amino acid substitutions have been shown to affect protein function and interfere with signaling [10], affect cellular localization [11], and impact on DNA and protein binding [12–14] in both plants and mammals. A role for NAGNAG alternative splicing was shown in human Stargardt disease [15] and has been implicated in other disease processes including cancer [16].

Large intergenic noncoding RNAs (lincRNAs) have traditionally been defined as long noncoding transcripts greater than 200 nucleotides in length. Overlapping isoforms of lincRNA have been reported previously and may include protein-coding genes [17]. Recently, while exploring the dynamic profiles of NAGNAG acceptors in Arabidopsis, we identified two isoforms originating from the same NAGNAG acceptors but located in noncoding RNA [18]. To date, previous studies have assumed NAGNAG acceptors function through the classical mRNA paradigm based on observation of altered coding for one or two amino acids in the protein-coding gene. Based on this observation of NAGNAG acceptors in Arabidopsis, we proposed an expanded paradigm and hypothesized that NAGNAG alternative splicing mechanism also exists in lincRNA.

Bioinformatics has become a powerful tool for the study of alternative splicing and its functional consequence. To date, bioinformatic analyses have produced evidence of alternative splicing in approximately 80% of human genes [19]. Bioinformatic approaches have been invaluable for exploring comparative genomics across species and such studies have produced important insights into regulatory mechanisms governing splicing and its role in evolution and adaptation. Single base-pair resolution offered by deep RNA sequencing motivated us to find further direct evidence of NAGNAG alternative splicing in lincRNA. To accomplish this goal we applied computational approaches to two public datasets of deeply-sequenced human tissue genomic data whose content included previously annotated lincRNA. By aligning the two RNA-seq data sets and systematically screening, identifying, and quantifying the NAGNAG alternative splicing of lincRNA, 31 NAGNAG alternative splicing events in lincRNA were defined. Importantly, tissue-specific patterns of expression for NAGNAG isoforms in lincRNA were observed.

## 2. Methods

### 2.1. Data

RNA-seq data sets were downloaded from NCBI SRA (accession number for data sets 1 and 2: E-MTAB-513 and GSE30554). These RNA-seq data were generated by sequencing 8 individual human tissues and mixture of 16 tissues (Illumina Body Map) using the Illumina HiSeq 2000 (Illumina, Inc.) platform. Each sample was deeply sequenced with more than 200 million reads and annotated for lincRNA. We only kept the high-quality reads using FastX quality filter with the following criteria: minimum of 20 Phred score over at least 80% of the sequence read.

### 2.2. Alignment, Screening, and Quantification

Annotations of human lincRNA were obtained from Human lincRNA Catalog hosted at Broad Institute [20]. All RNA-seq datasets were aligned to lincRNA with tophat [21] using the “-max-multihits 1”, which only permits unique mapping. The anchor length of the software was set at 8 nt and the mismatch number in these regions at 0 nt to avoid alignment bias. After the data were aligned, sequence postprocessing tool (SAMtools) was used to store, sort, and index the binary SAM data (bam files) with respect to sequence alignment (http://samtools.sourceforge.net) [22].

To identify lincRNA containing NAGNAG alternative splicing sites, we screened the lincRNA sequences using the classical expression of the “NAGNAG” motif. Alignment of RNA-seq reads to the NAGNAG splicing junctions was used to confirm and validate the existence of the splice sites. We required at least four junction reads with the same 5′ splice sites, stipulating that two needed to match the first NAGNAG splice site (site 1) while the other two were required to match the second NAGNAG splice site (site 2) [23, 24].

The sequences for splice sites and the 30 bp exonic and intronic flanking sequences were extracted based on hg19 genome sequence with Bioconductor package Biostrings (R package version 2.22.0). Sequence logos were drawn by WebLogo with default parameters as described previously [25]. Two flanking sequences of the NAGNAG acceptors, including 30 bp from intron and 30 bp from exon, were extracted and screened for the potential patterns. The ratio of isoform expression at two alternative splice sites (site 1 and site 2) was calculated as log(read counts at side 1*⁄*read counts at side 2). NAGNAG acceptors were grouped into four categories based on this ratio and the strand information. If the expression of isoform 1 was more than that of isoform 2, ratio > 0; otherwise, ratio < 0.

To quantify RNA expression levels, all RNA-seq counts were normalized using reads per million (RPM). The expression level of NAGNAG isoforms in lincRNA was calculated by read counts through Bioconductor package Rsamtools (R package version 1.6.3) and IRanges (R package version 1.12.6). Duplicate reads were kept for quantification purpose. NAGNAG motifs were only designated as NAGNAG acceptors if two splice sites exhibited more than 2 reads in at least two samples. To avoid ambiguity, we discarded those NAGNAG acceptors located in the overlapping area between lincRNAs and annotated genes.

### 2.3. Quantification of Tissue-Specific NAGNAG Acceptors

To analyze the relationship between the ratio of two NAGNAG splice sites and the tissues, we used Bioconductor package limma through the linear model:

(1) $$\begin{array}{c} Y_{ijk}=\alpha_{i}+\beta_{j}+\varepsilon_{ijk}, \end{array}$$

where *Y* represents the log ratio of two NAGNAG splice sites from the same NAGNAG acceptor, with NAGNAG acceptor *i*, tissue *j*, and sample *k*; *α* represents the main effect of *i*th NAGNAG acceptor; *β* represents the main effect of *j*th tissue; *ε* represents the measurement error. The NAGNAG acceptors were selected using false discovery rate (FDR)-adjusted *P* values < 0.05.

## 3. Results

Two novel observations were documented. First, mapping of unique reads to the potential NAGNAG alternative splicing sites in human lincRNA demonstrated existence of NAGNAG alternative splicing in lincRNA (Table 1). Of the 1320 lincRNAs containing the NAGNAG motif, presence of NAGNAG acceptors was confirmed with RNA-seq data in 30 lincRNAs. These 31 NAGNAG acceptors originate from 30 transcripts. Interestingly, linc-POLR3G-10 exhibited two NAGNAG acceptors located in two distinct transcripts: TCONS_00010012 and TCONS_00010010. Presence of two NAGNAG acceptors was identified in the upstream region of the fourth and fifth exons of this 5-exon gene. In addition, 8 NAGNAG acceptors were identified within the overlapping regions between lincRNA and protein-coding RNA but were not further considered in this study (see Supplementary Data 1 in Supplementary Material available online at http://dx.doi.org/10.1155/2014/736798).

**Figure 2:**
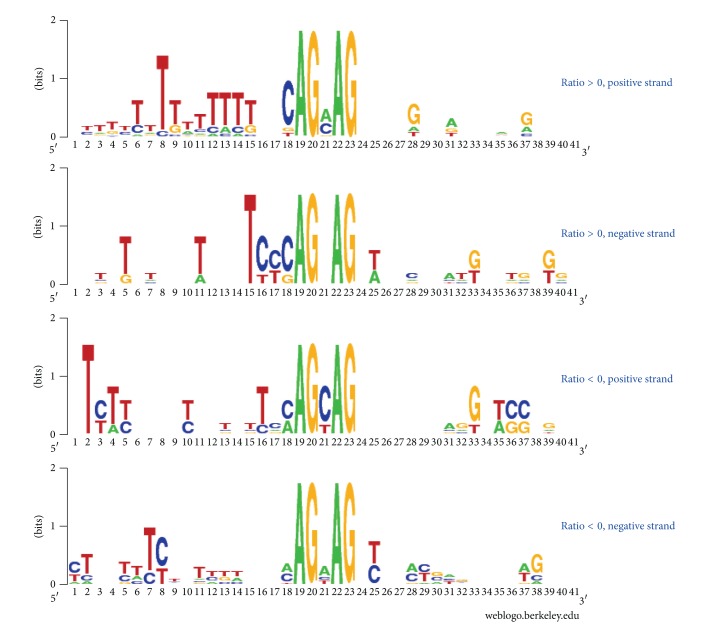
Sequence logos for 30 bp flanking sequences for 3′ splice sites. The logos are divided into four groups based on the chromosome strand and ratio of read counts of site 1 to site 2.

Most exons in lincRNA containing NAGNAG acceptors exceeded protein-coding genes in length (Wilcoxon rank sum test, *P* value < 2.2*e* − 16). The average exon length of protein-coding genes ranged between 306 ± 702 bp and the average neighbouring intron length ranged between 6092 ± 19983 bp (Supplementary Figure S1), compared to the average exon and intron length of lincRNA which ranged between 349 ± 630 bp and 8476 ± 19751 bp, respectively. Most tandem acceptors of lincRNA occurred at the furthest exon, that is, second exon occurring in the lincRNA (mean: 2.52; sd: 0.71) whereas those found in protein-coding genes were found centrally located among all of the exons occurring in the gene (mean: 10.7; sd: 8.8). The most prevalent triplet found among the lincRNA sequences was CAG for both splice sites, with GAG present at lowest frequency (Supplementary Table S1). CAGCAG and CAGAAG combinations occurred at highest frequency. Positive correlation with the expression level was found when CAG was encoded relative to splice site selection. Specifically, a predilection for the first splice site was noted when CAG was encoded at the first NAG site (ratio > 0, Figure 2). Alternatively, when CAG was located at the second NAG position or was absent from the splice site altogether, the second NAG was favoured for splicing (ratio < 0, Figure 2).

**Figure 3:**
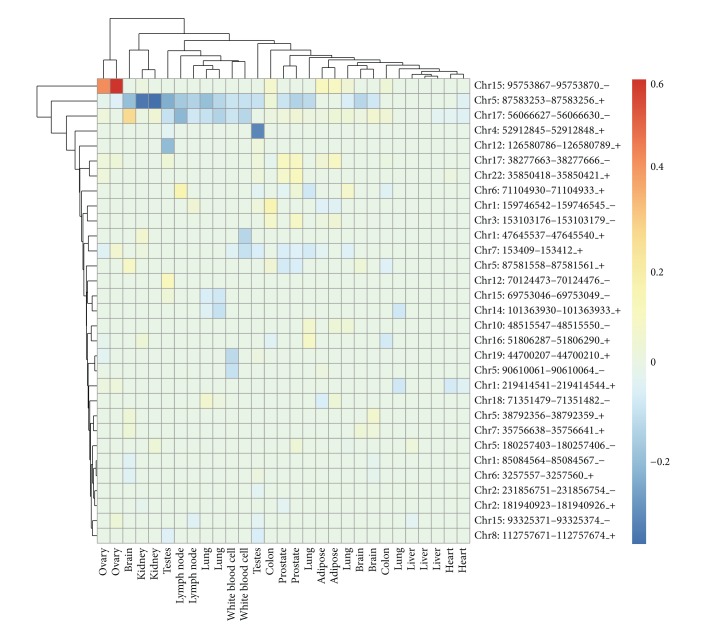
Heat map for the ratio of the NAGNAG isoforms at the two alternative splice sites (site 1 and site 2). Row represents 31 NAGNAG acceptors while column represents various tissues. Ratio > 0: site 2 is preferred. Ratio < 0: site 1 is preferred.

The second novel observation was demonstration of tissue-specific properties by 6 NAGNAG acceptors in lincRNA (FDR adjusted *P* value < 0.05).  Figure 3 shows that 6 of 31 NAGNAG acceptors exhibited statistically significant differences in expression levels across diverse tissues. Specifically, as seen in Figure 3, the first NAG splice site is specifically targeted by the NAGNAG acceptor: chr5:87583253-87583256_+ from TCONS_00010012. Presence of these splice sites was associated with a clear expression pattern in several tissues including lymph node, lung, and kidney, and this signature was remarkably consistent. Moreover, a similar pattern for the alternative splice sites was noted and the second NAG splice site was specifically targeted by NAGNAG acceptors: chr15:95753867-95753870_-. This distinctive expression pattern was clearly evident in ovary. Twenty-five of NAGNAG acceptors were notably absent or exhibited no difference in expression pattern across most tissues.

## 4. Discussion

Splice sites are pivotal factors in the splicing process [26]. NAGNAG alternative splicing was identified in the past decade and is characterized by inclusion or exclusion of three nucleotides at 3′ splice sites, resulting in substitutions in one or two amino acids in the protein products. Previous studies have shown that this type of alternative splicing is highly regulated and related to proteome evolution [1]. Functionally, NAGNAG alternative splicing in mRNA results in various isoforms which generate alternative proteins following translation.

To the best of our knowledge, the present study provides the first evidence that NAGNAG alternative splicing can be observed not only in mRNA but also in lincRNA. Although alternative splicing of lincRNA was reported previously [20], the report of NAGNAG alternative splicing is novel. Following analysis of two RNA-seq data sets including annotations for lincRNA, we identified 31 NAGNAG acceptors in lincRNA. These 31 NAGNAG acceptors originated from 30 transcripts. Interestingly, a role for “CAG” sequence was suggested in splice site selection with CAG being the most prevalent triplet found among the lincRNA sequences for both splice sites. GAG was present at lowest frequency and CAGCAG and CAGAAG combinations occurred at highest frequency. A predilection for the first splice site was noted when CAG was encoded at the first NAG site. The second NAG was favoured for splicing when CAG was located at the second NAG position or was absent altogether. This finding is consistent with the previous reports about mRNA [27].

Traditionally, lincRNA has been defined as stretches of DNA transcripts exceeding 200 base pairs in length which do not encode putative functional protein products [28]. lincRNA has been posited to play a role in splicing processes [29] and has been reported to contain predominately two exons [30]. In the current study, most exons from lincRNA containing NAGNAG acceptors exceeded protein-coding genes in length. Most tandem acceptors of lincRNA identified in the present study occurred at the furthest exon, that is, the second exon occurring in the lincRNA. By contrast those found in protein-coding genes have generally been found centrally located among all of the exons occurring in the gene.

The mechanism of this NAGNAG alternative splicing is not completely understood. Hiller and colleagues [3] suggested that these NAGNAG acceptors are not random noise because some fraction of NAGNAG acceptors is tissue-specific, although this theory was not universally shared by others [6, 8]. However, Bradley et al. provided solid evidence in support of tissue specificity based on RNA-seq analysis of 16 human and 8 mouse tissues wherein they demonstrated that at least 25% of NAGNAG acceptors in mRNA were regulated in a tissue-specific manner [1]. This percentage exceeded earlier estimates for tissue specificity [27]. Analysis of our selected datasets revealed low levels of consistent tissue-specific patterns relative to NAGNAG acceptors in lincRNA. Among 19% of NAGNAG acceptors that exhibited distinct differences in expression levels of certain tissues, targeting of specific splicing pattern among two NAGNAG acceptors was noted.

There are some limitations of this computational study. First, use of annotation data was limited to the Human lincRNA Catalog at Broad Institute [20], although other annotations of human lincRNA are also available [30]. More information about lincRNA will help to identify more NAGNAG alternative splicing. Second, biological significance and potential disease impact of NAGNAG alternative splicing was only projected computationally, and awaits confirmation through further proteomic studies. For example, results of gene ontology analysis by application for genes targeted by NAGNAG acceptors in lincRNA indicated that these genes were all functionally engaged in transcription regulation (ANP32A, CHD9, NR1D1, POLR3G, VEZF1, ZNF227) and signalling (CRP, CTBS, FAM174B, FAM20C, GDF10, ITGA4, NETO1, RGMA, TMEM132C, OSMR). Further, analysis for potential disease association of the neighbouring genes revealed that these genes represented candidate genes associated with risk for many important diseases, including hypertension, obesity, and cancer, among others (see Supplementary Table S2 for a complete list).

Importantly, bioinformatics analysis has proved to be an invaluable tool in the investigation of the role of alternative splicing from numerous perspectives including microarray analysis, alternative splicing prediction utilizing comparative genomic approaches, identification and depiction of isoform and splicing patterns, definition of regulation of alternative splicing, delineation of functional impact, and its role in defining evolutionary and adaptive processes, among other investigations [19]. To delineate alternative splicing in lincRNA, further investigations are essential in unraveling their functional and regulatory roles through application of bioinformatic, genetic, and proteomic approaches. The evolutionary aspect of lincRNA NAGNAG alternative splicing across different species can also be studied in the future.

## Supplementary Material

Supplementary Figure S1: The exon and intron length of protein-coding gene versus lincRNA.
Supplementary Table S1: Occurrence of NAGNAG acceptors in human long non-coding RNA.
Supplementary Table S2: Disease association of the lincRNA-neighboring genes.
Supplementary Data 1: lincRNA that overlap with protein-coding RNA.

## Figures and Tables

**Table 1:** NAGNAG acceptors in lincRNA confirmed by RNA-seq.

Transcript ID	linc name	chr	Site 1	Site 1 existence	Site 2	Site 2 existence	Strand	Neighbouring gene
TCONS_00000929	linc-CMPK1-3	chr1	47645537	Data 1, 2	47645540	Data 1, 2	+	CMPK1
TCONS_00001552	linc-CTBS-1	chr1	85084564	Data 1	85084567	Data 1	−	CTBS
TCONS_00002502	linc-CRP-1	chr1	159746542	Data 1	159746545	Data 1	−	CRP
TCONS_00002232	linc-IARS2-3	chr1	219414541	Data 1, 2	219414544	Data 1	+	IARS2
TCONS_00018502	linc-GDF10-1	chr10	48515547	Data 1	48515550	Data 1	−	GDF10
TCONS_00021357	linc-BEST3-1	chr12	70124473	Data 1	70124476	Data 2	−	BEST3
TCONS_00020623	linc-TMEM132C-14	chr12	126580786	Data 1, 2	126580789	Data 1	+	TMEM132C
TCONS_00023051	linc-DIO3-8	chr14	101363930	Data 2	101363933	Data 2	+	DIO3
TCONS_00023721	linc-ANP32A-1	chr15	69753046	Data 2	69753049	Data 1, 2	−	ANP32A
TCONS_00023791	linc-FAM174B-1	chr15	93325371	Data 1, 2	93325374	Data 1	−	FAM174B
TCONS_00023799	linc-RGMA-7	chr15	95753867	Data 1	95753870	Data 1	−	RGMA
TCONS_00024399	linc-CHD9-6	chr16	51806287	Data 1	51806290	Data 1	+	CHD9
TCONS_00025631	linc-NR1D1-1	chr17	38277663	Data 1	38277666	Data 1, 2	−	NR1D1
TCONS_00025146	linc-VEZF1-1	chr17	56066627	Data 1, 2	56066630	Data 1, 2	−	VEZF1
TCONS_00026560	linc-NETO1-1	chr18	71351479	Data 1, 2	71351482	Data 1, 2	−	NETO1
TCONS_00027051	linc-ZNF227-1	chr19	44700207	Data 1	44700210	Data 1	+	ZNF227
TCONS_00004960	linc-ITGA4-2	chr2	181940923	Data 1	181940926	Data 1	+	ITGA4
TCONS_00003507	linc-GPR55-1	chr2	231856751	Data 1, 2	231856754	Data 2	−	GPR55
TCONS_00029585	linc-RASD2-1	chr22	35850418	Data 1	35850421	Data 1	+	RASD2
TCONS_00005471	linc-TMEM14E-2	chr3	153103176	Data 1	153103179	Data 1	−	TMEM14E
TCONS_00007527	linc-SPATA18-1	chr4	52912845	Data 1, 2	52912848	Data 1, 2	+	SPATA18
TCONS_00009387	linc-OSMR-1	chr5	38792356	Data 1	38792359	Data 1	+	OSMR
TCONS_00010010	linc-POLR3G-10	chr5	87581558	Data 1, 2	87581561	Data 1, 2	+	POLR3G
TCONS_00010012	linc-POLR3G-10	chr5	87583253	Data 1, 2	87583256	Data 1	+	POLR3G
TCONS_00009724	linc-LYSMD3-2	chr5	90610061	Data 1, 2	90610064	Data 1, 2	−	LYSMD3
TCONS_00010581	linc-MGAT1-2	chr5	180257403	Data 1	180257406	Data 1	−	MGAT1
TCONS_00012396	linc-PSMG4-1	chr6	3257557	Data 1	3257560	Data 1	+	PSMG4
TCONS_00011322	linc-FAM135A-1	chr6	71104930	Data 1	71104933	Data 1	+	FAM135A
TCONS_00012862	linc-FAM20C-2	chr7	153409	Data 1	153412	Data 1	+	FAM20C
TCONS_00014103	linc-SEPT7-1	chr7	35756638	Data 1	35756641	Data 1, 2	+	SEPT7
TCONS_00014833	linc-UTP23-3	chr8	112757671	Data 1, 2	112757674	Data 2	+	UTP23
