# Supplementary material for: Computational Evidence of NAGNAG Alternative Splicing in Human Large Intergenic Noncoding RNA
Source: Biomed Res Int. 2014 Jun 5;2014:736798. doi: 10.1155/2014/736798 (PMC4068082; doi:10.1155/2014/736798)
Supplement: Supplementary file 1 — Supplementary Figure S1: The exon and intron length of protein-coding gene versus lincRNA. Supplementary Table S1: Occurrence of NAGNAG acceptors in human long non-coding RNA. Supplementary Table S2: Disease association of the lincRNA-neighboring genes. Supplementary Data 1: lincRNA that overlap with protein-coding RNA. [file 736798.f1.zip › 736798.f1/mat.736798.v3.doc]

Supplementary Figure for

**Computational evidence of NAGNAG alternative splicing in human long non-coding RNA**

Xiaoyong Sun^1^* , Simon M. Lin^2^*, Xiaoyan Yan^3^

^1^Agricultural Big-Data Research Center, College of Information Science and Engineering, Shandong Agricultural University, Taian, Shandong, 271018, P.R.China, ^2^Biomedical Informatics Research Center, Marshfield Clinic Research Foundation, Marshfield, WI 54449, USA, ^3^Affiliated Hospital of Shandong University of Traditional Chinese Medicine, No.42 Wenhua West Road, Jinan, Shandong, 250011, P.R.China

* To whom correspondence should be addressed. Tel: +086 538-8249346 Fax: +086 538- 8249275; Email: [johnsunx1@gmail.com](mailto:johnsunx1@gmail.com)

Correspondence may also be addressed to Simon M. Lin. Tel: +001 715-221-7299; Fax: +001 715-221-6402; Email: [LINMD.SIMON@mcrf.mfldclin.edu](mailto:LINMD.SIMON@mcrf.mfldclin.edu)

**Supplementary Figure S1.** The exon and intron length of protein-coding gene versus lincRNA

**Supplementary Figure S1.** The exon and intron length of protein-coding gene versus lincRNA

Supplementary Tables for

**Computational evidence of NAGNAG alternative splicing in human long non-coding RNA**

Xiaoyong Sun^1^* , Simon M. Lin^2^*, Xiaoyan Yan^3^

^1^Agricultural Big-Data Research Center, College of Information Science and Engineering, Shandong Agricultural University, Taian, Shandong, 271018, P.R.China, ^2^Biomedical Informatics Research Center, Marshfield Clinic Research Foundation, Marshfield, WI 54449, USA, ^3^Affiliated Hospital of Shandong University of Traditional Chinese Medicine, No.42 Wenhua West Road, Jinan, Shandong, 250011, P.R.China

* To whom correspondence should be addressed. Tel: +086 538-8249346 Fax: +086 538- 8249275; Email: [johnsunx1@gmail.com](mailto:johnsunx1@gmail.com)

Correspondence may also be addressed to Simon M. Lin. Tel: +001 715-221-7299; Fax: +001 715-221-6402; Email: [LINMD.SIMON@mcrf.mfldclin.edu](mailto:LINMD.SIMON@mcrf.mfldclin.edu)

**Supplementary Table S1.** Occurrence of NAGNAG acceptors in human long non-coding RNA

**Supplementary Table S2.** Disease association of the lincRNA-neighboring genes

**Supplementary Table 1.** Occurrence of NAGNAG acceptors in human long non-coding RNA

| **NAG position** | **Triplet** | **Occurrences** |
| --- | --- | --- |
|  | CAG | 21 |
| **First NAG** | AAG | 6 |
|  | TAG | 2 |
|  | GAG | 2 |
|  |  |  |
|  | CAG | 13 |
| **Second NAG** | AAG | 13 |
|  | TAG | 4 |
|  | GAG | 1 |
|  |  |  |
|  | CAGCAG | 10 |
|  | CAGAAG | 8 |
|  | AAGAAG | 3 |
|  | CAGTAG | 2 |
| **NAGNAG** | AAGTAG | 2 |
|  | GAGCAG | 2 |
|  | TAGAAG | 2 |
|  | AAGCAG | 1 |
|  | CAGGAG | 1 |

**Supplementary Table 2.** Disease association of the lincRNA-neighboring genes

| **Gene symbol** | **Disease name** |
| --- | --- |
| CMPK1 | malignant neoplasm of lung, cancer |
| CRP | disease by infectious agent  chronic rejection of renal transplant  heart disease  coronary heart disease  carcinoma  squamous cell carcinoma  atherosclerosis  cerebrovascular accident  myocardial infarction  acute myocardial infarction  pre-eclampsia  vascular disease  transient cerebral ischemia  ischemia  diabetes mellitus  polycystic ovary syndrome  syndrome  coronary stenosis  obesity  hyperinsulinism  liver cirrhosis  rheumatoid arthritis  arthritis  hypertension  gestational diabetes  heart failure  cancer  arteriosclerosis  dementia  Crohn's disease  kidney disease  chronic obstructive pulmonary disease  metabolic syndrome X  pulmonary embolism  lupus erythematosus  systemic lupus erythematosus  asthma  ankylosing spondylitis  spondylitis  periodontal disease  myeloma  colonic neoplasm  hepatocellular carcinoma  carotid stenosis  intermediate coronary syndrome  mucocutaneous lymph node syndrome  Cytomegalovirus infectious disease  adenoma  brain edema  malignant neoplasm of ovary  kidney failure  bipolar disease  medical disorder  scleroderma  systemic scleroderma  essential hypertension  hypercholesterolemia  arteriopathy  peritonitis  ulcerative colitis  macular degeneration  Kuhnt-Junius degeneration  age related macular degeneration  familial Mediterranean fever  brucellosis  antiphospholipid syndrome  cerebral infarction  abdominal aortic aneurysm  aneurysm  aortic aneurysm  vascular dementia  Alzheimer's disease  nephritis  pancreatitis  acute pancreatitis  glycogen storage disease  thrombocytopenia  periodontitis  dilated cardiomyopathy  leishmaniasis  visceral leishmaniasis  tuberculosis  Parkinson's disease  malaria  schizophrenia  brain infarction  Plasmodium falciparum malaria  pneumonia  hyperglycemia  multiple sclerosis  osteoarthritis  diabetic retinopathy  colorectal cancer  sarcoidosis  peptic ulcer  liver disease  fatty liver  myocardial ischemia  malignant neoplasm of breast  pericarditis  interstitial cystitis  cystitis  chronic interstitial cystitis  polycythemia  polycythemia vera  thrombocytosis  melanoma  pulmonary edema  newborn respiratory distress syndrome  adult respiratory distress syndrome  malignant neoplasm of lung  gestational trophoblastic neoplasm  chronic kidney failure  psoriasis  severe pre-eclampsia  diabetes mellitus type 2  neutropenia |
| DIO3 | chronic rejection of renal transplant  diabetes mellitus  osteoarthritis  carcinoma  hepatocellular carcinoma |
| ANP32A | retinoblastoma  cancer  osteoarthritis  chronic rejection of renal transplant |
| RGMA | cancer  malignant neoplasm of prostate  colorectal cancer |
| NR1D1 | \| atherosclerosis Osteoarthritis \| bipolar disease medical disorder \| cancer; \| malignant neoplasm of breast |
| NETO1 | schizophrenia |
| ITGA4 | chronic lymphocytic leukemia  leukemia  osteosarcoma  melanoma  carcinoma  myeloid leukemia  acute myeloid leukemia  multiple sclerosis  asthma  viral infectious disease  disease by infectious agent  rheumatoid arthritis  arthritis  macroglobulinemia  infertility  squamous cell carcinoma  autism  myeloma  multiple myeloma  in situ carcinoma  substance dependence  chronic rejection of renal transplant  syndrome  hemorrhagic fever with renal syndrome  RNA virus infectious disease |
| GPR55 | chronic rejection of renal transplant  cancer  malignant neoplasm of ovary  anorexia nervosa  obesity |
| RASD2 | chronic rejection of renal transplant |
| SPATA18 | muscular dystrophy  limb-girdle muscular dystrophy |
| OSMR | amyloidosis  cancer  carcinoma  squamous cell carcinoma  amyloidosis |
| MGAT1 | obesity |
| FAM20C | \| medical disorder syndrome |
| SEPT7 | Glioma chronic rejection of renal transplant schizophrenia carcinoma \| thyroid carcinoma; \| papillary thyroid carcinoma |

Supplementary Data for

**Computational evidence of NAGNAG alternative splicing in human long non-coding RNA**

Xiaoyong Sun^1,^*, and Simon M. Lin^2,^*

^1^ College of Information Science and Engineering, Shandong Agricultural University, Taian, Shandong, 271018, P.R.China.

^2^ Biomedical Informatics Research Center, Marshfield Clinic Research Foundation, Marshfield, WI 54449, USA.

* To whom correspondence should be addressed. Tel: +086 13153851616 Email: [johnsunx1@gmail.com](mailto:johnsunx1@gmail.com)
Correspondence may also be addressed to Simon M. Lin. Tel: +001 715-221-7299; Email: [LINMD.SIMON@mcrf.mfldclin.edu](mailto:LINMD.SIMON@mcrf.mfldclin.edu)

| ID | chr | site1 | site2 | strand | site1.TotalReadCount | site2.TotalReadCount | geneID | site1.Tissue | site1.Count | site2.Tissue | site2.Count |
| --- | --- | --- | --- | --- | --- | --- | --- | --- | --- | --- | --- |
| chr12:130639233-130639236_- | chr12 | 130639233 | 130639236 | - | 5 | 21 | gene_id XLOC_010263; transcript_id TCONS_00021000; exon_number 2; oId TCONS_00003296; linc_name linc-TMEM132D-1; tss_id TSS16097; class_code u; gene_name linc-TMEM132D-1; | 47y_ovary_female\|65y_lung_male_caucasian_paired | 2\|3 | 60y_adrenal_male\|65y_lung_male_caucasian_paired\|73y_adipose_female\|73y_prostate_male\|73y_prostate_male\|86y_lymphNode_female | 4\|6\|2\|2\|3\|4 |
| chr15:25363415-25363418_+ | chr15 | 25363415 | 25363418 | + | 787 | 96 | gene_id XLOC_011185; transcript_id TCONS_00023184; exon_number 2; oId NR_023915; linc_name linc-GABRA5-5; tss_id TSS17509; class_code .; gene_name linc-GABRA5-5; | 16tissueMix\|16tissueMix\|16tissueMix\|16tissueMix\|16tissueMix\|16tissueMix\|16tissueMix\|16tissueMix\|16tissueMix\|16tissueMix\|16tissueMix\|16tissueMix\|16tissueMix\|16tissueMix\|16tissueMix\|16tissueMix\|29y_breast_female_caucasian_paried\|37y_liver_male\|47y_ovary_female\|58y_whiteBloodCell_male\|60_kidney_female\|60y_adrenal_male\|60y_adrenal_male_paired\|60y_thyroid_female\|68y_colon_female\|68y_colon_female\|73y_prostate_male\|73y_prostate_male\|77y_brain_female\|77y_heart_male\|77y_heart_male\|77y_skeletalMuscle_male_caucasian_single\|brain\|Cultured_human_foreskin_fibroblast\|Cultured_human_lung_fibroblast\|Cultured_primary_human_lung_fibroblast\|Placenta\|testes | 27\|37\|32\|59\|52\|79\|32\|33\|64\|56\|8\|3\|5\|4\|7\|7\|4\|2\|7\|2\|22\|9\|3\|12\|8\|6\|2\|30\|8\|15\|34\|20\|7\|4\|30\|29\|3\|25 | 16tissueMix\|16tissueMix\|16tissueMix\|16tissueMix\|16tissueMix\|16tissueMix\|16tissueMix\|16tissueMix\|16tissueMix\|47y_ovary_female\|60y_adrenal_male\|60y_thyroid_female\|73y_adipose_female\|73y_prostate_male\|73y_prostate_male\|77y_heart_male\|77y_heart_male\|77y_skeletalMuscle_male_caucasian_single\|Cultured_human_lung_fibroblast\|Cultured_primary_human_lung_fibroblast\|testes | 3\|5\|2\|5\|3\|5\|3\|6\|2\|3\|6\|12\|4\|3\|3\|2\|6\|12\|2\|6\|3 |
| chr15:89931732-89931735_+ | chr15 | 89931732 | 89931735 | + | 154 | 71 | gene_id XLOC_011345; transcript_id TCONS_00023496; exon_number 3; oId TCONS_00004494; linc_name linc-C15orf42-2; tss_id TSS17757; class_code u; gene_name linc-C15orf42-2; | 16tissueMix\|16tissueMix\|16tissueMix\|16tissueMix\|16tissueMix\|16tissueMix\|16tissueMix\|16tissueMix\|16tissueMix\|16tissueMix\|16tissueMix\|16tissueMix\|16tissueMix\|16tissueMix\|16tissueMix\|16tissueMix\|19y_testes_male\|60y_thyroid_female\|77y_heart_male\|brain\|Placenta\|testes | 6\|9\|12\|10\|7\|4\|5\|12\|2\|8\|6\|8\|3\|3\|4\|5\|28\|2\|2\|2\|4\|12 | 16tissueMix\|16tissueMix\|16tissueMix\|16tissueMix\|16tissueMix\|16tissueMix\|16tissueMix\|16tissueMix\|16tissueMix\|16tissueMix\|16tissueMix\|16tissueMix\|16tissueMix\|16tissueMix\|19y_testes_male\|58y_whiteBloodCell_male\|77y_brain_female\|testes | 3\|4\|2\|2\|4\|2\|2\|4\|2\|4\|3\|5\|3\|3\|7\|9\|2\|10 |
| chr1:87823358-87823361_- | chr1 | 87823358 | 87823361 | - | 10 | 6 | gene_id XLOC_000901; transcript_id TCONS_00001559; exon_number 3; oId TCONS_00000855; linc_name linc-SEP15-3; tss_id TSS1387; class_code .; gene_name linc-SEP15-3; | 16tissueMix\|testes | 2\|8 | testes | 6 |
| chr2:49003419-49003422_+ | chr2 | 49003419 | 49003422 | + | 27 | 14 | gene_id XLOC_001466; transcript_id TCONS_00004786; exon_number 2; oId TCONS_00021619; linc_name linc-CHAC2-5; tss_id TSS2285; class_code u; gene_name linc-CHAC2-5; | 16tissueMix\|16tissueMix\|16tissueMix\|16tissueMix\|60y_thyroid_female\|68y_colon_female\|68y_colon_female\|77y_brain_female | 2\|2\|2\|3\|5\|3\|8\|2 | 68y_colon_female\|73y_prostate_male | 7\|7 |
| chr8:129108764-129108767_+ | chr8 | 129108764 | 129108767 | + | 200 | 4 | gene_id XLOC_006925; transcript_id TCONS_00014854; exon_number 2; oId TCONS_00013662; linc_name linc-EFR3A-4; tss_id TSS11134; class_code .; gene_name linc-EFR3A-4; | 16tissueMix\|16tissueMix\|16tissueMix\|16tissueMix\|16tissueMix\|16tissueMix\|16tissueMix\|16tissueMix\|16tissueMix\|16tissueMix\|16tissueMix\|16tissueMix\|47y_ovary_female\|47y_ovary_female_african\|58y_whiteBloodCell_male\|58y_whiteBloodCell_male\|60y_adrenal_male\|60y_adrenal_male_paired\|60y_thyroid_female\|73y_prostate_male\|73y_prostate_male\|86y_lymphNode_female\|Cultured_human_foreskin_fibroblast\|Cultured_human_lung_fibroblast\|Cultured_primary_human_lung_fibroblast | 12\|12\|5\|12\|18\|10\|2\|7\|5\|2\|2\|2\|3\|2\|14\|9\|12\|16\|6\|14\|3\|6\|2\|9\|15 | 19y_testes_male | 4 |
| chr15:95995024-95995027_+ | chr15 | 95995024 | 95995027 | + | 6 | 5 | gene_id XLOC_011368; transcript_id TCONS_00023531; exon_number 2; oId TCONS_00004530; linc_name linc-NR2F2-3; tss_id TSS17797; class_code u; gene_name linc-NR2F2-3; | 47y_ovary_female\|47y_ovary_female_african | 4\|2 | 16tissueMix\|16tissueMix | 2\|3 |
| chr20:23501384-23501387_+ | chr20 | 23501384 | 23501387 | + | 16 | 7 | gene_id XLOC_013491; transcript_id TCONS_00027833; exon_number 2; oId NR_001279; linc_name linc-TMEM90B-5; tss_id TSS21045; class_code u; gene_name linc-TMEM90B-5; | 19y_testes_male\|testes | 5\|11 | testes | 7 |
